# Big Five personality profiles, self-reported distress, and resilience resources among adults in Italy after the acute COVID-19 emergency: a cross-sectional cluster-analytic study

**DOI:** 10.3389/fpsyt.2026.1868864

**Published:** 2026-07-24

**Authors:** Tommaso Barlattani, Antony Bologna, Silvia Tamanti, Edoardo Trebbi, Valentina Socci, Alessandro Rossi, Rodolfo Rossi, Giorgio Di Lorenzo, Francesca Pacitti

**Affiliations:** 1 Department of Biotechnological and Applied Clinical Sciences (DISCAB), University of L’Aquila, L’Aquila, Italy; 2 Department of Public Health and Infectious Diseases, Sapienza University of Rome, Rome, Italy; 3 Department of Systems Medicine, University of Rome Tor Vergata, Rome, Italy; 4 Istituto di Ricovero e Cura a Carattere Scientifico (IRCCS) Fondazione Santa Lucia, Rome, Italy

**Keywords:** anxiety symptoms, Big Five, COVID-19, depressive symptoms, perceived stress, personality, post-emergency, resilience

## Abstract

**Introduction:**

Psychological recovery after the acute COVID-19 emergency has been uneven, and brief non-diagnostic markers that stratify vulnerability may support public mental health monitoring. We examined whether person-centered Big Five profiles differentiated self-reported distress and resilience resources among adults living in Italy after the end of the World Health Organization-declared COVID-19 public health emergency.

**Methods:**

This cross-sectional anonymous online survey included 2,169 adults assessed between 22 May 2023 and 23 December 2024. Participants completed the Big Five Inventory-10 (BFI-10), Generalized Anxiety Disorder-7 (GAD-7), Patient Health Questionnaire-9 (PHQ-9), Perceived Stress Scale-10 (PSS-10), and Resilience Scale for Adults-11 (RSA-11). Standardized Big Five scores were clustered using k-means, and the number of profiles was evaluated using elbow, silhouette, and hierarchical-clustering methods.

**Results:**

A two-profile solution was retained, although separation was modest (average silhouette width = 0.185). Compared with the resourceful trait profile (n = 1,113), the vulnerable trait profile (n = 1,056), characterized especially by lower emotional stability and conscientiousness, reported higher anxiety symptoms (g = 0.74), depressive symptoms (g = 0.76), and perceived stress (g = 0.86), and lower resilience resources (g = −1.02; all p < 0.001).

**Discussion:**

Brief person-centered Big Five profiling differentiated adults with greater self-reported symptom burden and fewer resilience resources in a prolonged post-emergency context. These findings are non-diagnostic and do not establish COVID-specific causal mechanisms, but may inform vulnerability stratification during future prolonged crises.

## 1 Introduction

The psychological consequences of COVID-19 have been substantial but have shown marked heterogeneity across individuals and across time. Early works documented increases in anxiety, depressive symptoms, and stress during the acute phases of the pandemic, while global burden estimates showed marked rises in depressive and anxiety disorders in 2020 (1–3). What remains less clear is how mental health trajectories have evolved during later phases, following the disruption of lockdowns and the formal end of the World Health Organization-declared Public Health Emergency of International Concern for the COVID-19 pandemic in May 2023 (4). Emerging syntheses focused on later and post-emergency phases indicate that the burden did not simply disappear but instead followed heterogeneous trajectories of recovery, persistence, and renewed vulnerability across settings and subgroups (5, 6). Longitudinal evidence points in the same direction. In Italy, overall distress declined 14 months after the first pandemic peak, yet a substantial minority still reported elevated self-reported symptoms (7). In Switzerland, depression, anxiety, and loneliness improved at the population level between 2021 and 2023, but higher-risk groups remained disadvantaged across follow-up (8). In a five-country European cohort assessed up to early 2024, symptoms varied with contextual pressures well beyond the acute pandemic period, including broader social and economic stressors (9). Together, these findings indicate that later-phase mental health cannot be reduced to a simple dichotomy between full recovery and continued crisis when considered at the aggregate level.

Italy remains an informative setting for examining this issue. A large body of Italian research has focused on the first lockdown and its immediate psychological impact, consistently identifying younger age and female gender as key correlates of worse mental health outcomes (10–12). However, the later context differed substantially from the acute-shock conditions of 2020. By 2023-2024, strict nationwide restrictions no longer defined everyday life, yet cumulative strain, financial pressures, disruptions to work and study routines, and lingering health concerns persisted.

Later evidence from Italy and other countries suggests that symptom reduction at the aggregate level can coexist with persistent burden in meaningful subgroups (5, 13, 14). From a public mental health perspective, this highlights the need to identify brief markers, beyond basic sociodemographic indicators, that can detect individuals who remain vulnerable and less resilient in post-emergency conditions.

Personality traits are plausible candidates because they shape appraisal, coping, self-regulation, and emotion regulation, and they show robust transdiagnostic links with internalizing psychopathology (15–17). Within the Big Five framework, lower emotional stability is consistently associated with anxiety and depressive symptoms, whereas conscientiousness, and often extraversion and agreeableness, tend to relate to better psychological adjustment (18, 19). During the acute pandemic, variable-centered studies repeatedly linked emotional instability to distress and identified conscientiousness and other self-regulatory tendencies as partial protective factors (20–25). Importantly, later-phase evidence suggests that these associations did not vanish with the easing of restrictions: neuroticism remained strongly related to depression, anxiety, and insomnia among university students in Wuhan in a later-phase context (26), and personality traits measured during the pandemic predicted adaptation five years later, with conscientiousness and extraversion linked to better longer-term adjustment (27).

From a diathesis-stress perspective, Big Five traits can be conceptualized as relatively stable dispositional vulnerabilities or resources that shape how individuals appraise, regulate, and cope with prolonged stressors (28). In a post-emergency context, residual pandemic-related concerns may coexist with broader sources of strain, including uncertainty, work or study disruption, economic pressure, and seasonal variation. Individuals with lower emotional stability may be more prone to threat appraisal, negative affect, and stress reactivity, whereas lower conscientiousness may be associated with weaker self-regulation, planning, and maintenance of adaptive routines. A person-centered Big Five approach may therefore help identify configurations of dispositional risk and resources that are relevant for non-diagnostic public mental health monitoring during prolonged post-acute or future crisis contexts.

Person-centered approaches address this issue by identifying recurring configurations of traits rather than isolated associations (29, 30). This approach may be particularly valuable when the aim is pragmatic stratification rather than examining associations at the level of individual traits.

In parallel, resilience resources remain highly relevant in later phases of collective stress. Resilience reflects protective individual and social capacities that support adaptation under adversity (31), is systematically related to personality, particularly emotional stability and conscientiousness (32), and has repeatedly been associated with better mental health during and after the pandemic (33–35). Later-phase data in students further suggest that coping profiles continue to differentiate distress and well-being after repeated lockdowns (36). Integrating symptom measures and resilience resources within a profile framework may therefore yield a psychologically meaningful map of current vulnerability.

Against this background, we used a person-centered approach to identify Big Five personality profiles in a large Italian community sample assessed during a prolonged post-emergency period after the acute COVID-19 emergency and tested whether profiles differed in self-reported anxiety symptoms, depressive symptoms, perceived stress, and resilience resources. We hypothesized that a profile characterized by lower emotional stability and conscientiousness would be associated with higher levels of distress and lower resilience resources relative to a more resourceful profile.

Identifying such profiles may have pragmatic relevance for public mental health. Brief, non-diagnostic personality profiling could support rapid vulnerability stratification during prolonged post-emergency or future crisis situations, helping to identify groups who may benefit from preventive outreach, psychoeducation, or low-intensity support.

## 2 Methods

### 2.1 Study design, participants, and ethics

This cross-sectional study analyzed data from an anonymous online survey conducted in Italy between 22 May 2023 and 23 December 2024, after the end of the World Health Organization-declared Public Health Emergency of International Concern for the COVID-19 pandemic on 5 May 2023 (4). This recruitment window should not be interpreted as a single homogeneous phase. Rather, it covered a prolonged post-emergency period during which residual pandemic-related concerns may have coexisted with seasonal variation, economic strain, and other contemporaneous social stressors.

The survey was developed in Google Forms^®^ and disseminated through sponsored social media posts; participants were also encouraged to share the link with acquaintances, resulting in a convenience and snowball sampling strategy. Participation was voluntary and uncompensated, and respondents could terminate the survey at any time.

Inclusion criteria were age ≥18 years, residence in Italy, and provision of written informed consent (online). The survey was implemented within a broader research program previously described by the group (37).

The initial dataset included 2,234 respondents. After data screening, 65 participants were excluded due to missing BFI-10 data, yielding a final analytic sample of N = 2,169.

Data were screened before analysis for eligibility and data quality. Screening included checks for age eligibility, residence in Italy, implausible values, and incomplete BFI-10 responses. No dedicated attention-check items were embedded in the survey. Because the survey was anonymous, IP addresses and device identifiers were not retained; therefore, duplicate participation could not be fully verified. Response time was not used as an exclusion criterion. These constraints are acknowledged as limitations of the online anonymous design.

Approval for the study was obtained from the local ethics committee/Institutional Review Board of the University of L’Aquila. The study was conducted in accordance with the Declaration of Helsinki.

All mental health measures were treated as self-reported symptom indicators and not as diagnostic assessments.

### 2.2 Measures

Sociodemographic variables included age, self-reported gender, education, relationship status, and presence of children. Gender was assessed as a self-reported sociodemographic variable and was not interpreted as biological sex. No analyses of biological sex were performed. Given the marked imbalance in reported gender, gender-related analyses were limited to descriptive and between-profile comparisons.

Personality traits were assessed using the Big Five Inventory-10 (BFI-10) (38), validated in Italian samples (39). The BFI-10 is an ultra-brief self-report measure of the Big Five framework and includes two items per trait rated on a 5-point Likert scale. It yields scores for Agreeableness, Conscientiousness, Emotional Stability (reverse Neuroticism), Extraversion, and Openness. In the present sample, the BFI-10 dimensions yielded low-to-moderate two-item reliability estimates, as expected for an ultra-short measure: Agreeableness inter-item r = 0.14, Spearman-Brown = 0.25; Conscientiousness r = 0.20, Spearman-Brown = 0.33; Emotional Stability r = 0.50, Spearman-Brown = 0.67; Extraversion r = 0.29, Spearman-Brown = 0.44; and Openness r = 0.20, Spearman-Brown = 0.34.

Anxiety symptoms were measured with the Generalized Anxiety Disorder-7 (GAD-7) (40), a 7-item self-report scale assessing the frequency of generalized anxiety symptoms over the previous two weeks on a 4-point Likert scale (0 = not at all, 3 = nearly every day). Scores are summed to obtain a total score ranging from 0 to 21, with higher scores indicating greater symptom severity. Conventional cut-offs of 5, 10, and 15 indicate mild, moderate, and severe symptom levels, respectively. Evidence from nationally representative European samples including Italy supports the unidimensionality, reliability, and measurement invariance of the GAD-7 (41). In the present sample, internal consistency was Cronbach’s α = 0.91 and McDonald’s ω = 0.92.

Depressive symptoms were measured with the Patient Health Questionnaire-9 (PHQ-9) (42), a 9-item self-report scale assessing depressive symptom frequency over the previous two weeks on a 4-point Likert scale (0 = not at all, 3 = nearly every day). The total score ranges from 0 to 27, with higher scores indicating greater depressive symptom severity. Conventional cut-offs of 5, 10, 15, and 20 indicate mild, moderate, moderately severe, and severe symptom levels, respectively. Evidence from nationally representative European samples including Italy supports the unidimensionality, reliability, and measurement invariance of the PHQ-9 (41). In the present sample, internal consistency was Cronbach’s α = 0.88 and McDonald’s ω = 0.89.

Perceived stress was measured with the Perceived Stress Scale-10 (PSS-10) (43), using the validated Italian version (44). The PSS-10 assesses the extent to which respondents perceived their life as unpredictable, uncontrollable, and overloaded during the previous month. Items are rated on a 5-point scale (0 = never, 4 = very often), with positively worded items reverse-scored according to standard procedures. Total scores range from 0 to 40, with higher scores indicating higher perceived stress. Although Italian validation work supports a two-factor structure, the total score was used in the present analyses. Internal consistency in the present sample was Cronbach’s α = 0.89 and McDonald’s ω = 0.89.

Resilience resources were measured with the 11-item Resilience Scale for Adults (RSA-11) (45), using the validated Italian brief version (46). The RSA-11 is rated on a 7-point scale and yields a total score ranging from 11 to 77. Higher scores indicate greater personal and interpersonal resilience resources. In the present sample, internal consistency was Cronbach’s α = 0.85 and McDonald’s ω = 0.85.

GAD-7 and PHQ-9 scores were interpreted as self-reported symptom severity indicators.

All measurement psychometric characteristics are reported in **Supplementary Table 1**.

### 2.3 Statistical analysis

Analyses were conducted using R (Version 4.5.1; 47) and RStudio (Version 2024.12.1+563; 48). Big Five scores were standardized (z-scores) before clustering. Personality profiles were derived using k-means clustering (49, 50). The optimal number of clusters was selected by triangulating the Elbow method (within-cluster sum of squares, WSS), average silhouette width (51), and inspection of hierarchical clustering output as a complementary check (52).

Missing data were handled by listwise exclusion for the variables required for the primary cluster solution. The initial dataset contained 2,234 respondents; 65 were excluded because BFI-10 data were missing, as complete Big Five scores were required for cluster assignment. No imputation was performed, and the outcome analyses were conducted on the final analytic sample of N = 2,169.

Because distributions departed from normality (53), between-profile differences in personality traits were evaluated using Wilcoxon rank-sum tests and summarized using effect size r. Categorical variables were compared using chi-square tests with Cramer’s V.

Associations between Big Five traits and outcomes were examined using Spearman correlations and interpreted using established guidelines (54). Differences between profiles across the combined outcome set (GAD-7, PHQ-9, PSS-10, RSA-11) were tested with MANOVA. Because multivariate assumptions were violated (including Box’s M test), Pillai’s trace was used. Significant multivariate effects were followed by univariate ANOVAs (55). Effect sizes included partial eta squared (η_p_^2^) and Hedges’ g with 95% confidence intervals. Statistical significance was set at α=0.05 (two-tailed).

Sensitivity analysis for time-related confounders. To address potential threats to internal validity arising from the extended recruitment window (May 2023-December 2024), recruitment time was operationalized both categorically (Period 1 = May-October 2023, Period 2 = November 2023-April 2024, Period 3 = May-December 2024) and continuously as months elapsed since study onset. We tested: (1) independence between cluster membership and recruitment period or season using chi-square tests and Cramer’s V; (2) outcome score differences across recruitment periods using Kruskal-Wallis tests; (3) a MANCOVA including continuous recruitment time as a covariate; and (4) visual stability of BFI-10 profiles across recruitment periods. These analyses are reported in **Supplementary Table 2** and **Supplementary Figure 1**.

## 3 Results

From 2,234 respondents, 65 were excluded due to missing BFI-10 data, leaving 2,169 participants for analysis. The mean age was 44.00 years (SD = 12.32). Among participants reporting gender, 82.5% were women. Sociodemographic characteristics by profile are reported in **Table 1**.

**Table 1:** Sociodemographic characteristics by personality profile.

Variable	Vulnerable (n=1056)	Resourceful (n=1113)	Test	p-value	Effect size
Age, years (M ± SD)	41.09 ± 12.10	46.77 ± 11.88	Mann-Whitney W = 431468	<0.001	–
Gender			χ^2^(2)=2.59	0.274	Cramer’s V = 0.035
Male	167 (16.1%)	205 (18.7%)			
Female	870 (83.9%)	889 (81.3%)			
Missing	19	19			
Education			χ^2^(4)=11.69	0.020	Cramer’s V = 0.073
Middle school	45 (4.3%)	54 (4.9%)			
High school diploma	383 (36.3%)	427 (38.5%)			
University degree	466 (44.2%)	419 (37.7%)			
Post-graduate degree	161 (15.3%)	210 (18.9%)			
Missing	1	3			
Relationship status			χ^2^(6)=59.55	<0.001	Cramer’s V = 0.166
Single	211 (20.0%)	162 (14.6%)			
In a relationship	204 (19.3%)	134 (12.1%)			
Cohabiting	240 (22.7%)	232 (20.9%)			
Married	328 (31.1%)	464 (41.8%)			
Separated/Divorced	62 (5.9%)	88 (7.9%)			
Widowed	6 (0.6%)	25 (2.3%)			
Missing	1	2			
Children			χ^2^(3)=51.52	<0.001	Cramer’s V = 0.154
None	641 (61.2%)	512 (46.3%)			
One	187 (17.9%)	245 (22.1%)			
More than one	219 (20.9%)	350 (31.6%)			
Missing	9	6			

Percentages are computed on non-missing responses within each profile.

**Figure 1:**
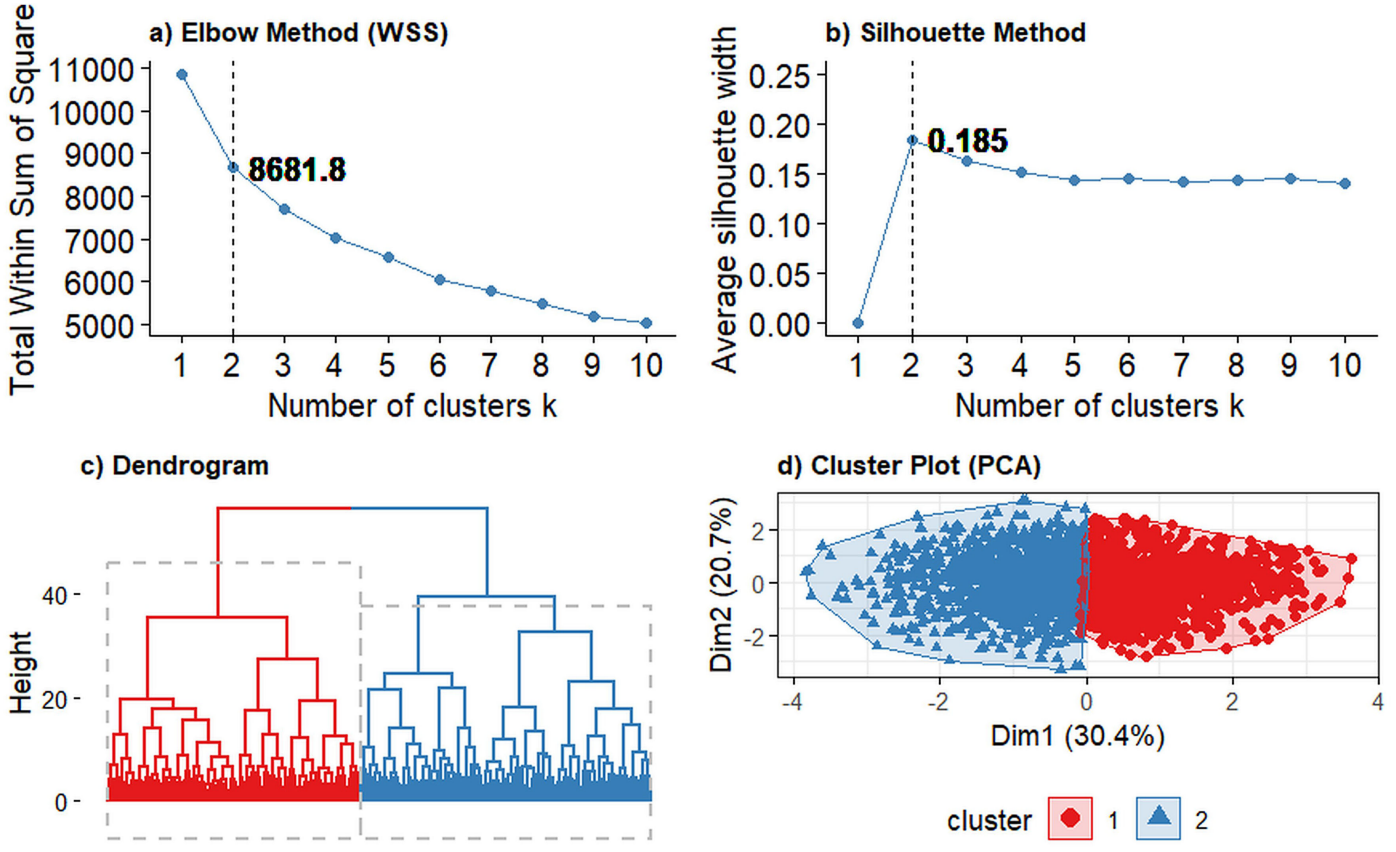
Determination of the optimal number of clusters (k) based on BFI-10 scores. **(A)** Elbow method: total within-cluster sum of squares (WSS) shows an inflection at k = 2 (WSS = 8681.8). **(B)** Silhouette method: average silhouette width peaks at k = 2 (0.185), supporting a two-profile solution with modest separation. **(C)** Dendrogram: hierarchical clustering suggests a primary bifurcation consistent with a two-profile structure. **(D)** Cluster plot (PCA): projection onto the first two principal components shows partial overlap, expected when clustering continuous personality dimensions.

Internal indices supported a two-profile solution. The Elbow method showed an inflection at k=2 (WSS = 8681.8), and the silhouette method yielded the highest average silhouette width for k=2 (0.185), indicating modest but maximal separation. Hierarchical clustering suggested a primary bifurcation consistent with two profiles (**Figure 1**). We therefore retained a two-profile solution.

**Figure 2:**
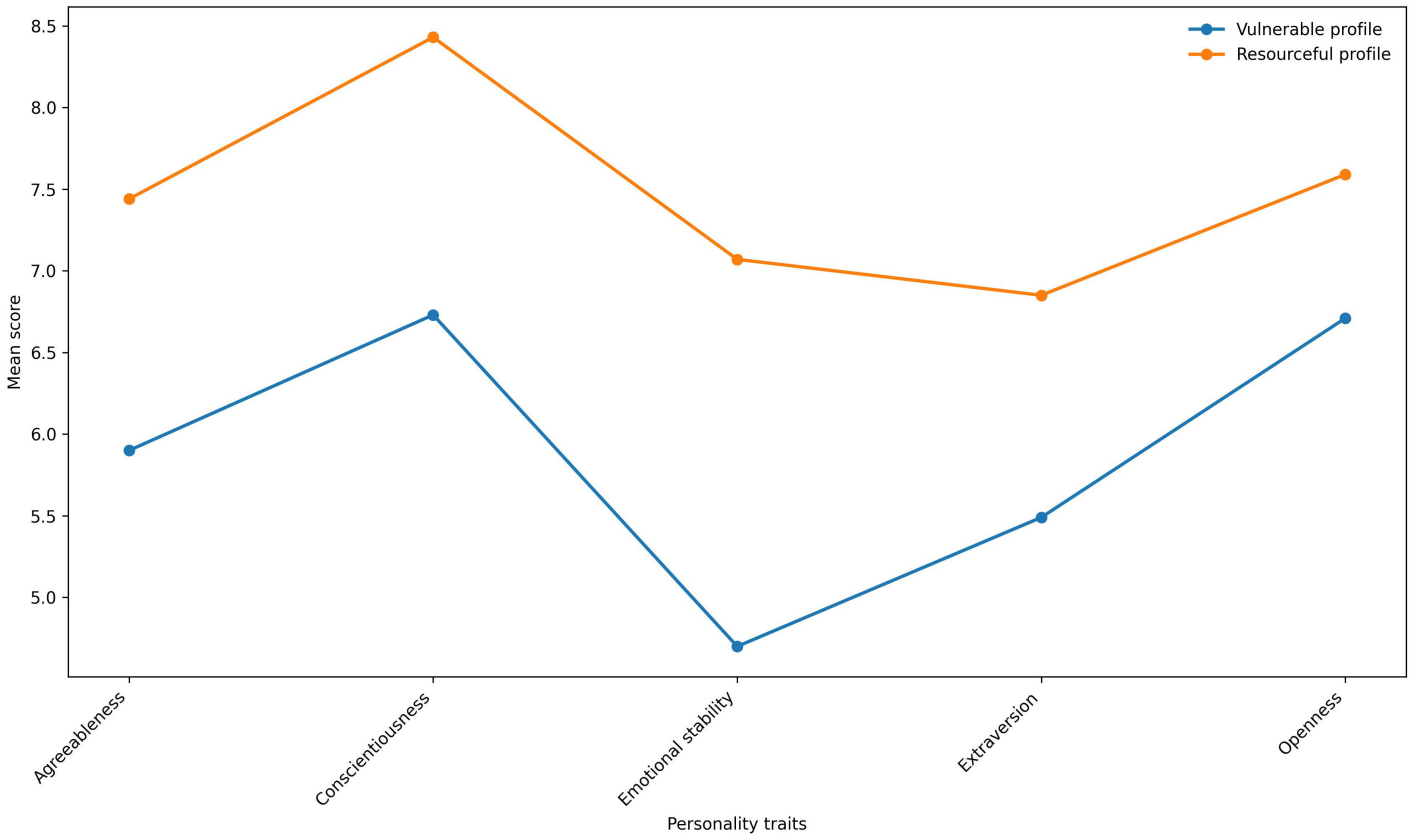
Mean BFI-10 trait scores by personality profile. Comparison of mean scores across the BFI-10 dimensions in the vulnerable and resourceful personality profiles.

K-means clustering identified two profiles: a vulnerable trait profile (n=1,056; 48.7%) and a resourceful trait profile (n=1,113; 51.3%), accounting for 19.9% of the total variance. The vulnerable profile showed lower levels across all traits, with the largest differences in emotional stability (r=0.57) and conscientiousness (r=0.53) (**Table 2**, **Figure 2**). These labels are used for interpretive clarity and do not imply categorical personality “types”.

**Table 2:** Big Five personality traits by personality profile (BFI-10 sum scores).

Trait	Vulnerable M (SD)	Resourceful M (SD)	Wilcoxon W	p-value	Effect size r	Magnitude
Agreeableness	5.90 (1.57)	7.44 (1.40)	278438	<0.001	0.46	Moderate
Conscientiousness	6.73 (1.48)	8.43 (1.28)	234026	<0.001	0.53	Large
Emotional stability	4.70 (1.71)	7.07 (1.75)	201971	<0.001	0.57	Large
Extraversion	5.49 (1.72)	6.85 (1.66)	341726	<0.001	0.37	Moderate
Openness	6.71 (2.03)	7.59 (1.82)	442959	<0.001	0.22	Small

Profiles differed on several sociodemographic indicators. Participants in the vulnerable trait profile were younger than those in the resourceful trait profile (41.09±12.10 vs 46.77±11.88 years, p<0.001). Gender distribution did not differ significantly (p=0.274). Education, relationship status, and presence of children also showed significant profile differences (**Table 1**).

**Figure 3:**
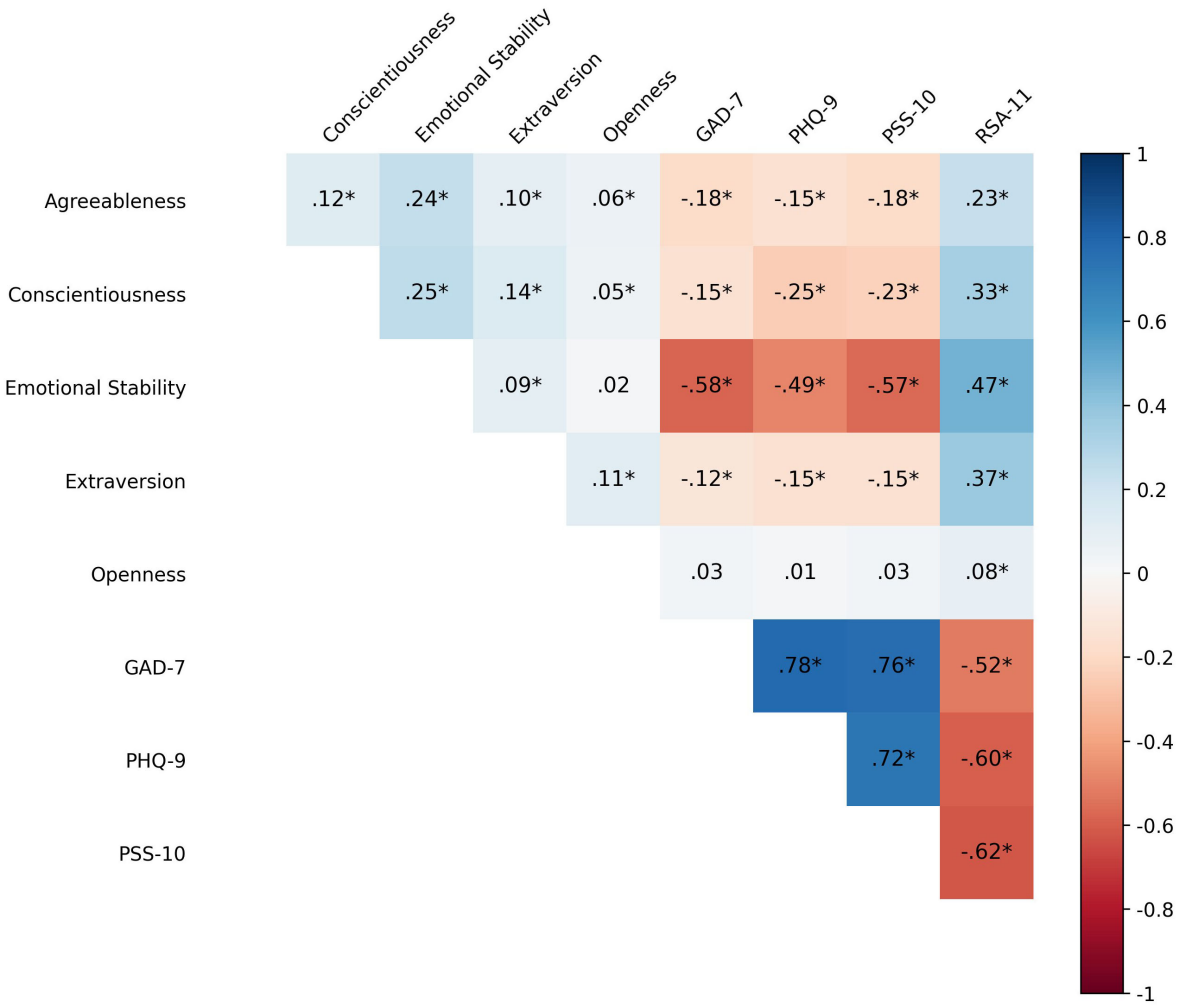
Spearman correlation matrix. Spearman correlation matrix between Big Five dimensions (BFI-10) and outcomes (GAD-7, PHQ-9, PSS-10, RSA-11). *p < 0.05.

Across the full sample, traits correlated in the expected directions with distress and resilience (**Figure 3**). Emotional stability showed the strongest associations with anxiety symptom scores (r_s_=-0.58), depressive symptoms (r_s_=-0.49), perceived stress (r_s_=-0.57), and resilience resources (r_s_=0.47).

MANOVA confirmed that profile membership was strongly associated with the combined outcome set (Pillai’s trace=0.23; F(4,2164)=161.6; p<0.001). Univariate models showed consistent differences across all outcomes (**Table 3**). Compared with the resourceful trait profile, the vulnerable trait profile reported higher anxiety symptom scores (g=0.74), higher depressive symptoms (g=0.76), higher perceived stress (g=0.86), and markedly lower resilience resources (g=-1.02) (all p<0.001).

**Table 3:** MANOVA and univariate outcome differences by personality profile (Pillai’s trace = 0.23; F(4,2164) = 161.6; p < 0.001).

Outcome	Vulnerable M (SD)	Resourceful M (SD)	F(1,2167)	p-value	η_p_^2^	Hedges’ g	95% CI
GAD-7	8.98 (5.28)	5.28 (4.66)	298.80	<0.001	0.12	0.74	0.66 to 0.83
PHQ-9	9.19 (5.77)	5.29 (4.42)	315.34	<0.001	0.13	0.76	0.67 to 0.85
PSS-10	20.94 (7.37)	14.70 (7.18)	399.98	<0.001	0.16	0.86	0.77 to 0.95
RSA-11	46.68 (12.30)	58.67 (11.22)	562.69	<0.001	0.21	-1.02	-1.11 to -0.93

Hedges’ g is computed as (Vulnerable - Resourceful); negative values indicate lower scores in the vulnerable profile.

Temporal sensitivity analyses. Although the formal recruitment window extended from May 2023 to December 2024, 2,160 of 2,169 participants (99.6%) completed the baseline assessment between May and October 2023. The remaining participants were recruited across the following 14 months (Period 2: n = 8; Period 3: n = 1). Cluster membership was independent of recruitment period (χ^2^(2) = 1.67, p = .434, Cramer’s V = .028) and season (χ^2^(3) = 1.86, p = .602, V = .029). Outcome scores did not differ across recruitment periods for GAD-7, PHQ-9, PSS-10, or RSA-11 (all p >.34, all η^2^H <.001). When continuous recruitment time was entered as a covariate in MANCOVA, the multivariate cluster effect remained unchanged (Pillai’s Trace = .234, F(4, 2163) = 164.93, p <.001, η^2^p = .23), whereas recruitment time was non-significant (Pillai = .002, p = .375; ΔPillai vs. original MANOVA = .000). Visual inspection of BFI-10 profiles confirmed the expected vulnerable-resourceful distinction in Period 1; Periods 2 and 3 were not interpretable as independent replications because of their negligible sample sizes. Overall, the cluster solution and profile-outcome associations were not attributable to recruitment timing.

## 4 Discussion

In this large Italian community sample assessed during a prolonged post-emergency period after the acute COVID-19 emergency, a brief person-centered Big Five approach identified two near-equally sized profiles associated with clearly different mental health patterns. The vulnerable profile—defined most strongly by lower emotional stability and conscientiousness—reported higher anxiety symptom scores, depressive symptom scores, and perceived stress, together with markedly lower resilience resources. The effect sizes were medium-to-large for distress outcomes and large for resilience, suggesting that even an ultra-brief trait measure captured psychologically meaningful heterogeneity.

The prominence of emotional stability and conscientiousness is theoretically coherent. Lower emotional stability reflects greater negative affectivity and stress reactivity, which may increase vulnerability to anxiety symptoms, depressive symptoms, and perceived stress. Lower conscientiousness may reflect weaker self-regulation, planning, persistence, and maintenance of adaptive routines, all of which are relevant when individuals face prolonged uncertainty or cumulative stress. Within a diathesis-stress framework, these two traits may therefore represent complementary pathways of vulnerability: one related primarily to affective reactivity and the other to regulatory capacity.

These findings align with established models linking emotional instability to internalizing symptoms and stress reactivity, and conscientiousness to planning, self-regulation, and adaptive coping (16, 17). They also extend earlier COVID-19 findings by showing that dispositional vulnerability remains relevant within a later, post-emergency context rather than only at the onset of lockdowns (23, 26, 27). This matters because later-phase mental health appears uneven rather than uniformly recovered. Recent Italian, Swiss, and broader European data suggest that average improvement can coexist with persistent burden in vulnerable subgroups and with renewed stress arising from changing contextual pressures (7–9). Our findings are consistent with this pattern, identifying a large subgroup characterized by worse self-reported outcomes across multiple domains.

A key strength of the study is the joint consideration of symptoms and resilience resources. Resilience is not simply the absence of symptoms; it captures protective capacities that support adaptation under adversity (31). The lower RSA-11 scores observed in the vulnerable profile indicate fewer self-reported resilience resources at the time of assessment. This finding should not be interpreted as evidence that these individuals will necessarily show poorer future adaptation, because the cross-sectional design does not establish temporal order. However, it suggests that personality profiles and resilience resources may be jointly informative for identifying individuals who report greater current vulnerability. This interpretation is consistent with work linking personality to resilience and with later-phase studies showing that coping and resilience continue to differentiate outcomes after the acute pandemic period (32, 34–36).

At the same time, the findings should not be read as evidence of fixed personality “types” or COVID-specific causal pathways. The two-cluster solution was the best-supported among the tested alternatives, but the average silhouette width (0.185) indicates modest separation. This is not unusual in large community datasets using brief trait measures, yet it means the profiles should be interpreted as useful empirical groupings rather than sharply bounded categories. In addition, the long recruitment window spanning mid-2023 to late-2024 may have introduced time-related influences. Participants completed the survey across different seasons and across a period in which residual pandemic-related concerns may have coexisted with economic pressures, health concerns, and other societal stressors. Therefore, the findings are best interpreted as describing associations observed in a prolonged post-emergency context rather than isolating mechanisms uniquely attributable to COVID-19.

The BFI-10 reliability estimates warrant caution. The Spearman-Brown coefficients for the two-item trait indices ranged from 0.25 to 0.67, consistent with the bandwidth-fidelity trade-off of ultra-short instruments (38, 56). The low BFI-10 coefficients should therefore be interpreted as a limitation of measurement precision rather than as evidence that the remaining outcome measures were unreliable; by contrast, GAD-7, PHQ-9, PSS-10, and RSA-11 showed high internal consistency in this sample.

Several limitations warrant emphasis. The cross-sectional design precludes conclusions about temporal ordering or causal direction. Therefore, we cannot determine whether personality profiles preceded distress and resilience differences, whether current distress influenced self-reported personality responses, or whether both were shaped by unmeasured contextual factors. The online convenience/snowball sample, with strong female over-representation, limits generalizability. Although participants reported gender, we did not perform gender-stratified analyses, which further limits the generalizability of the findings. The BFI-10 is efficient but sacrifices precision compared with longer trait measures. No structured psychiatric diagnostic interview, clinician-rated assessment, neurological examination, neurocognitive assessment, or medical-record verification was performed. Accordingly, GAD-7 and PHQ-9 scores should be interpreted as self-reported symptom severity indicators rather than psychiatric diagnoses. The study also did not assess neurological disorders, neurocognitive symptoms, long-COVID manifestations, SARS-CoV-2 infection history, hospitalization, bereavement, or other COVID-specific exposures. Therefore, the findings cannot be used to estimate the prevalence of psychiatric or neurological disorders and cannot attribute the observed profile differences specifically to COVID-19 infection or neuropsychiatric sequelae. The extended recruitment window may have introduced seasonal and contextual variation, and we were unable to model whether profile-outcome associations varied over the 2023–2024 collection period. Finally, although the profile solution showed coherent external validity, cluster separation was modest. Replication with more representative samples, richer trait measures, diagnostic and medical information where appropriate, and explicitly time-sensitive analyses is needed.

The online anonymous design also limited data quality controls. No dedicated attention-check items were included, and the absence of retained IP addresses, device identifiers, or response-time exclusion criteria prevented full verification of duplicate participation or careless responding.

Despite these limitations, the study provides evidence that brief person-centered Big Five profiling can differentiate a large subgroup with substantially worse self-reported distress and markedly fewer resilience resources within a prolonged post-emergency period. These findings do not demonstrate COVID-specific causal mechanisms, but they may inform future public mental health strategies. In future pandemics or collective crises, brief non-diagnostic personality profiling may complement symptom monitoring by helping identify groups who could benefit from preventive outreach, psychoeducation, and low-intensity support.

## 5 Conclusions

Within a prolonged post-emergency period in Italy, brief person-centered Big Five profiling differentiated adults with higher self-reported anxiety symptoms, depressive symptoms, perceived stress, and lower resilience resources. These findings do not demonstrate COVID-specific causal mechanisms and should not be interpreted as psychiatric diagnoses. However, they suggest that non-diagnostic personality profiling may complement symptom monitoring and preventive outreach during future pandemics or collective crises.

## Data Availability

The raw data supporting the conclusions of this article will be made available by the authors, without undue reservation.

## Supplementary material

Supplementary Table 1 Reliability indices and score ranges for study measures. For the BFI-10, inter-item correlations and Spearman-Brown coefficients are reported because each Big Five trait is assessed by two items. Cronbach’s α and McDonald’s ω are reported for multi-item scales.

Supplementary Table 2 Sensitivity analysis: tests of temporal confounding across recruitment periods. Recruitment periods: Period 1 = May-October 2023 (n = 2,160); Period 2 = November 2023-April 2024 (n = 8); Period 3 = May-December 2024 (n = 1). SA1: χ^2^ tests of independence between cluster membership and temporal variables; Cramer’s V as effect size. SA2: Kruskal-Wallis tests on outcome scores across recruitment periods; η^2^H = H/(N-1). SA3: MANCOVA with continuous recruitment time (months from study onset) as covariate; η^2^p = partial eta squared. ΔPillai between original MANOVA and time-adjusted MANCOVA = 0.000. MANCOVA, multivariate analysis of covariance.

Supplementary Figure 1 Sensitivity analyses of temporal confounding. Recruitment distribution, cluster membership proportions, and BFI-10 profile stability across recruitment periods. The recruitment distribution shows that 2,160 of 2,169 participants (99.6%) completed the assessment during May-October 2023. Cluster membership was independent of recruitment period (χ^2^(2) = 1.67, p = .434, Cramer’s V = .028). Outcome scores did not differ significantly across periods (all p >.34, η_H_^2^ <.001), and continuous recruitment time was non-significant in the MANCOVA (p = .375; ΔPillai = 0.000). Cluster 1 = Vulnerable profile; Cluster 2 = Resourceful profile.
