# Supplementary material for: Big Five personality profiles, self-reported distress, and resilience resources among adults in Italy after the acute COVID-19 emergency: a cross-sectional cluster-analytic study
Source: Front Psychiatry. 2026 Jul 24;17:1868864. doi: 10.3389/fpsyt.2026.1868864 (PMC13448549; doi:10.3389/fpsyt.2026.1868864)
Supplement: Supplementary file 1 [file Supplementaryfile1.docx]

**Supplementary Table S1. Reliability indices and score ranges for study measures.**

| **Measure / trait** | **Items** | **Score range** | **Cronbach's alpha** | **McDonald's omega** | **Inter-item r** | **Spearman-Brown coefficient** |
| --- | --- | --- | --- | --- | --- | --- |
| BFI-10 Agreeableness | 2 | 2-10 | - | - | 0.14 | 0.25 |
| BFI-10 Conscientiousness | 2 | 2-10 | - | - | 0.20 | 0.33 |
| BFI-10 Emotional stability | 2 | 2-10 | - | - | 0.50 | 0.67 |
| BFI-10 Extraversion | 2 | 2-10 | - | - | 0.29 | 0.44 |
| BFI-10 Openness | 2 | 2-10 | - | - | 0.20 | 0.34 |
| GAD-7 | 7 | 0-21 | 0.91 | 0.92 | - | - |
| PHQ-9 | 9 | 0-27 | 0.88 | 0.89 | - | - |
| PSS-10 | 10 | 0-40 | 0.89 | 0.89 | - | - |
| RSA-11 | 11 | 11-77 | 0.85 | 0.85 | - | - |

Note. For the BFI-10, inter-item correlations and Spearman-Brown coefficients are reported because each Big Five trait is assessed by two items. Cronbach's alpha and McDonald's omega are reported for multi-item scales.

**Supplementary Table S2. Sensitivity analysis: tests of temporal confounding across recruitment periods.**

| **Analysis** | **Statistic** | **F / H** | **p** | **Effect size** |
| --- | --- | --- | --- | --- |
| **A) Cluster membership independence** |  |  |  |  |
| Cluster x Recruitment Period | χ²(2) = 1.67 | — | .434 | V = .028 |
| Cluster x Season | χ²(3) = 1.86 | — | .602 | V = .029 |
| **B) Outcome scores across recruitment periods (Kruskal-Wallis)** |  |  |  |  |
| GAD-7 | H(2) = 0.53 | — | .768 | η²H < .001 |
| PHQ-9 | H(2) = 2.09 | — | .351 | η²H < .001 |
| PSS-10 | H(2) = 2.14 | — | .343 | η²H < .001 |
| RSA-11 | H(2) = 1.66 | — | .437 | η²H < .001 |
| **C) MANCOVA controlling for continuous recruitment time** |  |  |  |  |
| Cluster effect | Pillai = .234 | F(4, 2163) = 164.93 | < .001 | η²p = .23 |
| Recruitment time (covariate) | Pillai = .002 | F(4, 2163) = 1.06 | .375 | η²p = .002 |

Note. Recruitment periods: Period 1 = May-October 2023 (n = 2,160); Period 2 = November 2023-April 2024 (n = 8); Period 3 = May-December 2024 (n = 1). A: chi-square tests of independence between cluster membership and temporal variables; Cramer's V as effect size. B: Kruskal-Wallis tests on outcome scores across recruitment periods; η²H = H/(N-1). C: MANCOVA with continuous recruitment time (months from study onset) as covariate; η²p = partial eta squared. ΔPillai between original MANOVA and time-adjusted MANCOVA = 0.000. MANCOVA = multivariate analysis of covariance.

**Supplementary Figure S1. Sensitivity analyses of temporal confounding**

Recruitment distribution, cluster membership proportions, and BFI-10 profile stability across recruitment periods. The recruitment distribution shows that 2,160 of 2,169 participants (99.6%) completed the assessment during May-October 2023. Cluster membership was independent of recruitment period (χ²(2) = 1.67, p = .434, Cramer's V = .028). Outcome scores did not differ significantly across periods (all p > .34, η²H < .001), and continuous recruitment time was non-significant in the MANCOVA (p = .375; ΔPillai = 0.000). Cluster 1 = Vulnerable profile; Cluster 2 = Resourceful profile.
